# Cache a Killer: Cache Valley virus seropositivity and associated farm management risk factors in sheep in Ontario, Canada

**DOI:** 10.1371/journal.pone.0290443

**Published:** 2023-08-24

**Authors:** Michele D. Bergevin, Victoria Ng, Paula Menzies, Antoinette Ludwig, Samira Mubareka, Katie M. Clow

**Affiliations:** 1 Population Medicine, University of Guelph, Guelph, Ontario, Canada; 2 National Microbiology Laboratory Branch, Public Health Agency of Canada, Guelph, Ontario, Canada; 3 National Microbiology Laboratory Branch, Public Health Agency of Canada, St. Hyacinthe, Quebec, Canada; 4 Sunnybrook Health Research Institute, Toronto, Ontario, Canada; 5 Department of Laboratory Medicine and Pathology, University of Toronto, Ontario, Canada; Universidad Santo Tomas, CHILE

## Abstract

Cache Valley virus (CVV) disease is a mosquito-borne zoonosis endemic to North America. CVV disease is reported most often in sheep, causing lethal congenital deformities. There are limited data on CVV in Ontario, which is the largest sheep producing province in Canada. This study aimed to determine CVV seroprevalence in Ontario sheep flocks and investigate farm management factors associated with CVV exposure. A cross-sectional study was performed including 364 mature ewes across 18 farms selected from the five largest sheep districts in the province. A questionnaire was administered at each farm to determine farm management practices pertinent to the flock and ewes specifically sampled. Mixed multivariable logistic regression with a random effect for farm was conducted to assess associations between CVV seropositivity (outcome variable) and farm management risk factors (predictor variables). CVV seroprevalence was 33.2% in individual ewes (95% CI: 28.4%-38.1%) as determined by a virus neutralization assay with a titre > 4. Sixteen of the eighteen flocks (88.9%) had at least one CVV seropositive ewe. Increased age, smaller flock size, and sheep housing near wetlands, lakes, or ponds were found to be significantly associated with higher odds of CVV seropositivity. These findings are valuable in guiding breeding practices and housing during mosquito season to minimize infection and, ultimately, CVV disease in the flock.

## Introduction

Cache Valley virus_(CVV) of the *Orthobunyavirus* genus and Bunyamwera serogroup (BUN) is mosquito-borne and endemic to North America [1, 2]. CVV disease affects both humans and animals but is mostly reported and studied in sheep, where it often presents as lethal congenital deformities [3, 4]. Pre-weaning mortality rates in sheep typically range from 10% to 30% in Canada, with stillbirths and death within the first three days of life representing the majority of lamb mortalities [5–7]. The cause of most newborn mortalities are not determined, and of the minority cases that are diagnosed, 18% are associated with congenital abnormalities, dystocia, or an undetermined infectious agent [7]. Given that CVV disease results in similar clinical outcomes, it warrants further investigation as a contributing factor to losses in early life.

In North America, CVV has been isolated from over 40 mosquito species [2, 8–10], of which 30 are established in Ontario [11–13]. However, the role these mosquito species play in viral transmission is still unknown, and vector competency studies are needed to further elucidate the significance of these findings [13]. Seasonally, CVV is detected in mosquitoes between July and October, with peak viral load occurring in August and September, based on field studies in the northeastern United States [8, 13–17]. To achieve desired weight points in lambs for New Year’s holidays when market demand is significantly higher than other times of the calendar year, it is a common practice in Ontario to breed sheep in late summer to lamb in December and January [18]. Consequently, early gestation coincides with peak viral activity in mosquitoes. If ewes are bred during this high-risk period prior to being exposed to infected mosquitoes and developing CVV antibodies that protect fetuses in early gestation, they are at greater risk of becoming infected and delivering CVV diseased lambs during the winter lambing season [19].

While it may not be feasible for shepherds to shift breeding schedules, there may be other farm management practices that, when modified, could ultimately reduce risk of CVV infection to their flocks during critical breeding periods. Recently, numerous CVV outbreaks have been reported in Ontario sheep flocks, but contributing factors remain unknown [20–22]. Further, no studies have explored risk factors associated with CVV seropositivity. Meyers et al. explored the effect of farm management practices on BUN exposure in flocks across the USA and identified age, flock size and housing as significant indicators of seropositivity, although neither the magnitude of association nor the specific contribution from CVV was investigated [23]. Additional studies are needed to corroborate these findings, especially in the Canadian context. The objectives of this study were two-fold: to evaluate the flock-level and ewe-level seroprevalence of CVV infection in southern Ontario, and to identify farm management practices and environmental exposures that increase or mitigate risk of CVV exposure in sheep.

## Methods

### Study design

A cross-sectional study design was utilized to estimate flock and ewe level seroprevalence and assess risk factors. A sample size calculation for proportions was used with an *a priori* seroprevalence estimate of 20% with 95% confidence and 10% margin of error. The sample size was adjusted for clustering at the farm-level, using an intraclass correlation coefficient (ICC) of 0.25 and 20 ewes per farm, which yielded 357 ewes across 18 farms [24, 25]. To evaluate risk factor associations, a sample size calculation to estimate the difference between proportions was used. The *a priori* seroprevalence estimates for each group were set at 40% and 10% (with and without the proposed risk factor, respectively), with 95% confidence and 80% power. The sample size was adjusted for clustering using an ICC of 0.25 and 20 ewes per farm, which yielded 184 ewes per group (368 total) on 20 farms.

This study was approved by the University of Guelph Research Ethics Board (REB) and the Animal Care Committee (AUP) for compliance with federal guidelines for research involving human participants (REB#20-01-024) and standards mandated by the Canadian Council on Animal Care (AUP#4361), respectively.

### Farm recruitment

The five largest sheep districts in Ontario (districts 2, 3, 5, 6, and 7), based on total number of ewes, were chosen for farm recruitment [26]. A combination of random and convenience sampling for farm recruitment was implemented. Initially, the Ontario Sheep Farmers (OSF), a non-profit organization that promotes, supports, and advocates for the sheep industry, randomly identified and solicited by mail for study participation 20 farms in each of the five districts. Shepherds were eligible to participate if they had at least 20 mature ewes that had given birth at least once and had been on the farm prior to the previous summer (June 2020) to ensure a minimum of one full season of potential exposure to mosquitoes. Shepherds were asked to return the response form in the prepaid envelope included with the solicitation letter regardless of whether they chose to participate, and to provide a reason if they declined. A second round of recruitment was initiated three months later, whereby the chairs of each district were contacted and asked to redistribute the solicitation letter using social media avenues and word of mouth typically used to communicate news to their district’s OSF members. Shepherds who agreed to participate, and that met the eligibility criteria, were enrolled in the study on a first-come first-serve basis until 18 farms were secured.

### Risk factor questionnaire

Prior to visiting the farms, a questionnaire was conducted via telephone between a member of the research team and the shepherd overseeing the flock at each farm. The same researcher conducted all surveys for consistency. The questionnaire was provided to shepherds in advance either via email or mail (S1 File). Responses were recorded over the phone while conducting the questionnaire live. Upon arrival to collect field samples, questionnaire answers were reviewed in person between the same individuals to ensure accurate recording of the original responses or to make necessary changes. Questionnaire topics included: demographics; flock medical history; management practices including housing, seasonal movement, breeding schedule; self-identified surrounding environmental factors; and insecticide/repellent use. Location of lambing, breeding, and any movement of sheep off-farm (e.g., grazing) were captured as well, to properly assess whether farm sites where sheep were sampled fully represented individual exposure risk.

### Serum collection and CVV seroprevalence testing

Jugular blood samples were collected from 20 ewes per farm using a vacutainer, 10 mL red top non-serum separator tubes, and 20-gauge 1 inch venipuncture needles. If a flock had more than 20 ewes, a subset of ewes was selected by guiding ewes through a chute and sampling every *N*^*th*^ ewe where *N* equated to the quotient of the flock and sample sizes to ensure ewe selection was equal regardless of chronological order through the chute. Samples were stored on ice prior to same day processing in the laboratory. Extracted serum was stored at -20°C prior to being shipped frozen to Texas A&M Veterinary Medical Diagnostics Laboratory (College Station, TX) for CVV antibody testing using a virus neutralization assay (S2 File, [27, 28]). Titres > 4 were considered positive for exposure to CVV.

### Statistical analysis

Descriptive statistics were calculated for risk factors identified from the questionnaire, and sparse categories were collapsed for statistical analysis. A causal diagram guided the identification of risk factors for regression analysis (S3 File).

Mixed logistic regression with a random intercept for farm was conducted based on the dichotomous outcome of individual ewe CVV seropositivity (Yes / No). Risk factors identified from the causal diagram were first screened using univariable analysis, and those with liberal significance (*α* = 0.20) were included in the multivariable model. Continuous risk factors were evaluated for linearity and transformed or categorized if necessary to fulfill model assumptions. Linearity was assessed graphically by plotting a locally weighted regression of the outcome on the continuous risk factor (lowess) and examining the smoothed line for straightness. Linearity was also assessed statistically by generating a quadratic term of the continuous risk factor in the regression model and examining statistical significance of the term (*α* = 0.05) [25]. Collinearity between risk factors was evaluated using Spearman’s rank correlation and if greater than |0.80|, correlated variables were analyzed in separate models. Based on the causal diagram, risk factors thought to be confounders were also included in the initial multivariable regression analysis. Interaction was investigated between biologically plausible variables that had sufficient sample sizes.

The association of housing during mosquito season and CVV infection status was of particular interest and was therefore evaluated in two ways in separate models: 1) an index that weighted different housing structures according to mosquito exposure risk and 2) dummy variables that evaluated each housing option individually. The weighted housing variable ranged from 0.0 to 1.0 and was calculated in the following manner to quantify mosquito exposure per season (June–October) based on housing. Low exposure = 0.0 for closed barn, mid-exposure = 0.5 for open barn/drylot housing, high exposure = 1.0 for time spent in pasture. Exposure values per ewe were weighted by time of day (daytime vs. nighttime) and portion of a single season within each housing locale. For example, the mosquito exposure index for an ewe that spends June-July in pasture during daytime and drylot during nighttime, and is moved into barn for August-October, would be calculated as follows, *Mosquito exposure index =*

$$\begin{array}{l} {\left(\frac{2}{5}\right)}_{June,\;July\;}*\;\left(\left({\frac{1}{2}}_{Day}*1.0_{Pasture\;}\right)\;+\;\left({\frac{1}{2}}_{Night}*0.5_{Drylot\;}\right)\right)\;\\ +\;{\left(\frac{3}{5}\right)}_{Aug,\;Sept,\;Oct\;}*\;\left(\left({\frac{1}{2}}_{Day}*\;0.0_{Barn\;}\right)\;+\;\left({\frac{1}{2}}_{Night}\;*\;0.0_{Barn\;}\right)\right) \end{array}$$

which equates to 0.30. Note that in cases where housing changed based on age, the index was adjusted proportional to age such that the final calculation represented a single season.

Next, all risk factors that passed screening via univariable analysis were included in multivariable analysis that applied manual backward stepwise selection and a more stringent significance threshold (*α* = 0.05) to create the final model. Prior to removing individual non-significant risk factors from the multivariable mixed model, they were evaluated for confounding based on a 25% difference between crude and adjusted coefficient values. Likelihood ratio tests were applied prior to removing non-significant categorical risk factors from the final model via manual selection. To validate results from the manual analysis, automated backward stepwise selection accounting for clustering at the farm level was also evaluated, using a significance level for removal and inclusion of *α* = 0.20 and 0.05, respectively from the model. Non-nested models were compared using the Akaike’s information criterion (AIC), where the lowest AIC value identified the best fit model. Finally, Pearson’s residuals were calculated to evaluate outliers, while normality and homoscedasticity of the Best Linear Unbiased Predictors (BLUPs) were examined to ensure model assumptions were met [25]. All statistical analysis was performed in Stata IC 16.1 (StataCorp, College Station, Texas, USA).

## Results

### Farm recruitment

After rigorous recruitment efforts, eighteen farms agreed to participate in the study. Recruitment strategies secured farms via the original recruitment letter (n = 3), OSF weekly newsletters (n = 7), a rural county newspaper article describing the study (n = 3), and non-profit social media websites aimed at sheep producers (n = 5). Shepherds originally solicited by mail that provided a reason for declining to participate stated either ineligibility due to insufficient flock size (n = 10) or no longer farming (n = 8).

### Demographics and descriptive statistics

**Fig 1:**
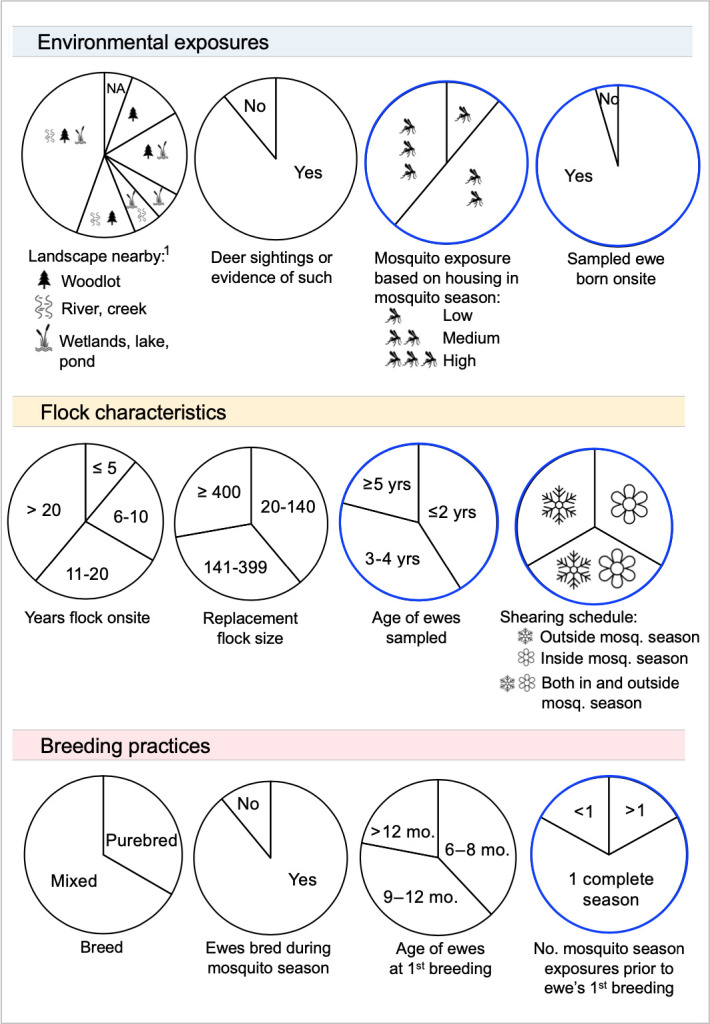
Summary demographic information for ewes sampled (n = 364) and their representative flocks, based on data obtained from the questionnaire, which was answered in full by each of the 18 shepherds. The blue and black circles indicate ewe-level and flock-level questions, respectively. Note: mosquito season refers to June through October, based on monthly surveillance of mosquitoes by Public Health Ontario [29]. ^1^Nearby is defined as within 1 km, which represents the distance that mosquitoes fly between breeding and feeding habitats [30].

Summary demographic information about the ewes (n = 364) and farms (n = 18) was obtained from the questionnaire that each shepherd answered in its entirety (Fig 1 and S1 File). The median age of sampled ewes was 3.3 years and ranged from 1.2 years to 10.3 years. All flocks were raised for the purpose of meat except for one dairy farm. Most flocks were cross-breeds with no one breed predominating in this study. The median flock size was 188 breeding ewes, and the range was from 24 to 840 ewes. Farm management experience also varied: 2–5 years of experience managing sheep (n = 2), 6–10 years (n = 4), 11–20 years (n = 5), and over 20 years of experience (n = 7). These durations of experience also aligned with the number of years that flocks had been on their respective farms. All but one farm reported additional livestock onsite, but the species and quantity varied greatly across farms, preventing statistical analysis.

Eleven shepherds reported a history of CVV disease in their flock during at least one season based on an official diagnostic test (n = 6) or assessment of the classic clinical signs of disease including lordosis, scoliosis, or arthrogryposis (n = 5). Two farms bred ewes outside of mosquito season and neither reported a history of CVV disease in their flocks. For the remaining 16 farms, the percentage of flocks that experienced CVV disease increased as the age decreased at which ewe-lambs were first bred. Specifically, at farms where ewe-lambs were first bred: at 6–8 months old, four out of five flocks experienced CVV disease (80%); at 9–12 months old, five out of seven flocks experienced CVV disease (71%); and at >12 months old, two out of four flocks experienced CVV disease (50%).

No sampled ewes had temporarily traveled off the farm, but 20 ewes were born off site. Six ewes arrived onsite prior to their first mosquito season exposure. Three ewes experienced only their first mosquito season elsewhere, of which two were six years old and one was nine years old at sampling. The 11 remaining ewes born offsite arrived in Ontario mid-August of their first mosquito season, all of which were three years old at sampling. Therefore, the regions where ewes were sampled sufficiently captured their exposure risk to mosquitoes.

Evidence of deer across the farms was ubiquitous, and no sheep producers used insecticide targeting mosquitoes on the sheep nor near housing structures. Several farms did use fly deterrents near animal housing structures. Descriptive statistics were performed on individual and farm-level characteristics obtained from the questionnaire (Table 1).

**Table 1:** Descriptive statistics of individual and farm-level characteristics (n = 364).

RISK FACTOR	CATEGORY		
**CATEGORICAL**		**No. ewes per category (% total ewes)**	**No. CVV+ ewes (% in the category)**
***Environmental exposures***			
Woodlot nearby^a^	Yes	304 (83.5%)	101 (33.2%)
	No	60 (16.5%)	20 (33.3%)
Wetlands, lake, or pond nearby^a^	Yes	262 (72.0%)	111 (42.4%)
	No	102 (28.0%)	10 (9.8%)
River or creek nearby^a^	Yes	224 (61.5%)	86 (38.4%)
	No	140 (38.5%)	35 (25.0%)
***Housing effect on mosquito exposure***			
Closed barn ^b^	Yes, but not necessarily exclusive	179 (49.2%)	45 (25.1%)
	No	185 (50.8%)	76 (41.1%)
Pasture ^b^	Yes, but not necessarily exclusive	344 (94.5%)	119 (34.6%)
	No	20 (5.5%)	2 (10.0%)
Confined outdoor housing, (e.g., drylot or open barn) ^b^	Yes, but not necessarily exclusive	140 (38.5%)	74 (52.9%)
	No	224 (61.5%)	47 (21.0%)
Mosquito exposure index based on housing, weighted by time of day, month of season, and age; ranging from 0.0–1.0	Low (0.00–0.50)	166 (45.6%)	27 (16.3%)
	Low—Medium (0.51–0.63)	50 (13.7%)	35 (70.0%)
	Medium—High (0.64–0.77)	60 (16.5%)	35 (58.3%)
	High (0.78–1.00)	88 (24.2%)	24 (27.3%)
***Flock & Ewe demographics***			
Flock size (replacement ewes)	20–140	141 (38.7%)	64 (45.4%)
	141–399	120 (33.0%)	40 (33.3%)
	≥ 400	103 (28.3%)	17 (16.5%)
Years flock onsite	≤ 5	42 (11.5%)	13 (31.0%)
	6–10	80 (22.0%)	4 (5.0%)
	11–20	102 (28.0%)	37 (36.3%)
	> 20	140 (38.5%)	67 (47.9%)
Breed	Mixed	243 (66.8%)	65 (26.7%)
	Purebred	121 (33.2%)	56 (46.3%)
Shearing schedule for ewe	Outside mosquito season (Nov—May)	173 (47.5%)	60 (34.7%)
	During mosquito season (Jun—Oct)	128 (35.2%)	49 (38.3%)
	Both	63 (17.3%)	12 (19.0%)
***Breeding characteristics***			
Breeding frequency of flock	Once per year	201 (55.2%)	84 (41.8%)
	Multiple times per year	163 (44.8%)	37 (22.7%)
Age ewe-lamb first bred	6–8 months old	139 (38.2%)	44 (31.7%)
	9–12 months old	145 (39.8%)	21 (14.5%)
	> 12 months old	80 (22.0%)	56 (70.0%)
***Medical history of flock***			
CVV disease history in flock	No	142 (39.0%)	38 (26.8%)
	Yes: lab test or classic signs observed	222 (61.0%)	83 (37.4%)
**CONTINUOUS**		**n (%)**	**Median (Range)**
***Mosquito exposure***			
Age (Years)	Ewe _**CVV+**_	121 (33.2%)	4.3 (1.3–10.3)
	Ewe _**CVV–**_	243 (66.8%)	2.9 (1.2–8.4)

^**a**^Indicates natural landscapes within 1 km of ewe housing at any time during the year.

^**b**^Event must have occurred between June and October. Eligible timeframe is based on seasonal CVV-vector activity [8, 13, 14, 16, 17].

### CVV seroprevalence

**Fig 2:**
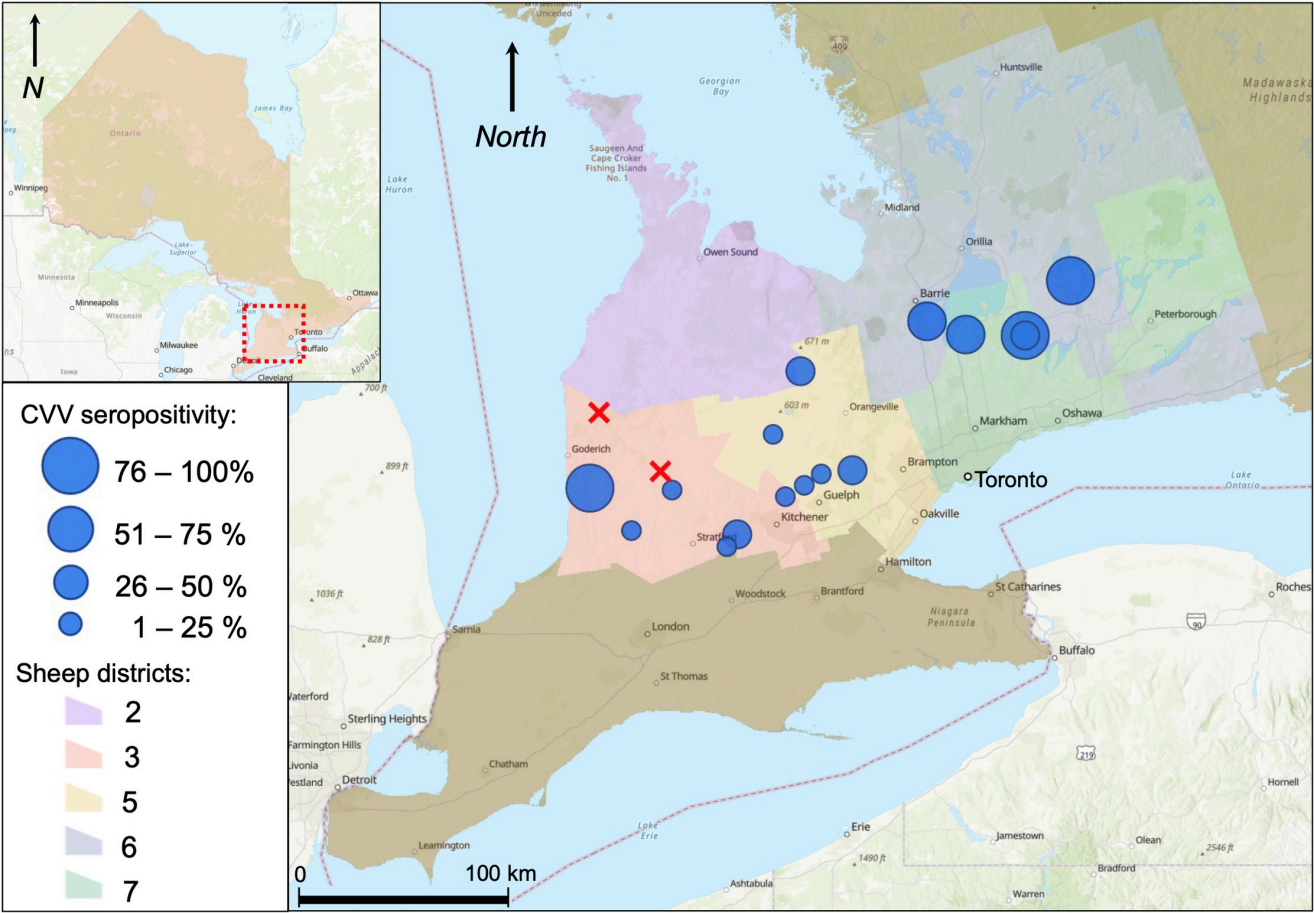
Sixteen sheep farms sampled across five sheep districts in southern Ontario hadewes that were seropositive for CVV (blue circles). Ewes sampled at two farms were all seronegative (red crosses). Basemap permitted by ESRI (ArcGIS Online, Redlands, California) [31]. Sheep districts defined by OSF [32].

Sample collection occurred from June 14 through August 17, 2021, with only two farms sampled in August. Twenty ewes were sampled at each locale except three farms, where 19 ewes were sampled at one farm, and at two farms, two and three extra ewes were sampled, respectively due to initial concern of insufficient serum volumes collected from originally selected ewes. The extra samples ultimately proved unnecessary but were still included in analysis. In total, 364 individual ewe samples were tested for CVV neutralizing antibodies. CVV prevalence at the ewe-level was 33.2% (121/364, 95% CI: 28.4%-38.1%). At the flock level, 88.9% (16/18, 95% CI: 72.8%-100.0%) had ≥ 1 seropositive ewe. Among the CVV seropositive flocks, the median was 35.0% seropositivity (7/20 seropositive ewes per flock), the range was 5.0% to 90.0% (1/20 to 18/20) seropositive ewes. The CVV antibody titres ranged from 8 to 2048 with a median of 256. At the two farms where CVV was not detected, ewes sampled were all ≤ 2 years old. CVV seropositivity was widespread across the sampling region (Fig 2). Eight of the 20 ewes born offsite were CVV seropositive, but based on the provided history, exposure likely occurred onsite. Three of these ewes arrived onsite prior to the first mosquito season. Three ewes arrived onsite in August at 10 months old and were sampled at age 3. The final two ewes were six and nine years old at sampling and experienced only the first full mosquito season offsite.

### Mixed logistic regression

Several risk factors had to be modified from their original state to fulfill model assumptions prior to univariable analysis (Table 2). Specifically, age remained a continuous predictor, but flock size and mosquito exposure risk had to be categorized.

**Table 2:** Risk factors associated with CVV seropositivity in sheep based on univariable mixed logistic regression with a random effect for farm of serological and questionnaire data obtained from sheep producers in Ontario, Canada (June–August 2021, n = 364).

RISK FACTOR	CATEGORY	OR (95% CI)	P-value
**CATEGORICAL**			
***Environmental exposures***			
Woodlot nearby^a^	Yes	0.73 (0.07–7.97)	0.793
	No	Referent	–
Wetlands, lake, or pond nearby^a^	Yes	7.27 (1.25–42.21)	0.027
	No	Referent	–
River or creek nearby^a^	Yes	1.64 (0.27–10.13)	0.592
	No	Referent	–
***Housing***			
Closed barn^b^	Yes, but not necessarily exclusive	0.31 (0.06–1.74)	0.184
	No	Referent	–
Pasture^b^	Yes, but not necessarily exclusive	4.05 (0.08–197.10)	0.480
	No	Referent	–
Confined outdoor housing, (e.g., drylot or open barn)^b^	Yes, but not necessarily exclusive	7.36 (1.48–36.51)	0.015
	No	Referent	–
Mosquito exposure index based on housing, weighted by time of day, month of season, and age; ranging from 0.0–1.0	Low (0.00–0.50)	Referent	–
	Low–Medium (0.51–0.63)	28.46 (5.03–160.87)	0.000
	Medium–High (0.64–0.77)	17.64 (3.28–94.97)	0.001
	High (0.78–1.00)	1.96 (0.31–12.49)	0.476
***Flock & Ewe demographics***			
Flock size (replacement ewes)	20–140	8.70 (1.02–73.95)	0.048
	141–399	4.10 (0.45–37.35)	0.211
	≥ 400	Referent	–
Years flock onsite	≤ 5	Referent	–
	6–10	0.09 (0.01–1.38)	0.083
	11–20	1.15 (0.10–3.85)	0.911
	> 20	2.30 (0.21–24.69)	0.493
Breed	Mixed	Referent	–
	Purebred	3.07 (0.50–19.01)	0.227
Shearing schedule for ewe	Outside mosquito season (Nov–May)	Referent	–
	During mosquito season (Jun–Oct)	1.17 (0.19–7.27)	0.868
	Both	3.27 (0.57–18.64)	0.182
***Breeding characteristics***			
Breeding frequency of flock	Once per year	Referent	–
	Multiple times per year	0.21 (0.04–1.22)	0.083
Age ewe-lamb first bred	6–8 months old	Referent	–
	9–12 months old	0.29 (0.07–1.16)	0.079
	> 12 months old	7.94 (1.37–46.03)	0.021
***Medical history of flock***			
CVV disease history in flock	No	Referent	–
	Yes: lab test or classic signs observed	1.83 (0.29–11.45))	0.517
**CONTINUOUS**			
Age at sampling	Per year increment	1.66 (1.36–2.03)	<0.001

^**a**^Indicates natural landscapes within 1 km of ewe housing at any time during the year.

^**b**^Event must have occurred between June and October. Eligible timeframe is based on seasonal CVV-vector activity [8, 13, 14, 16, 17].

Ewe housing during mosquito season was highly variable across farms and therefore it was difficult to adequately categorize this variable. When assessed as a dummy variable, sheep that spent time in confined outdoor housing in the summer or fall season had higher odds of being CVV seropositive (OR: 7.4, 95% CI: 1.5–36.5). Sheep that spent time in closed barn housing in the summer or fall had reduced odds of CVV exposure (OR: 0.3, 95% CI: 0.1–1.7) (Table 2). None of the housing dummy variables remained significant in multivariable analysis. When housing was assessed as a weighted variable to illustrate potential mosquito exposure over the season (June through October), the low-medium and medium-high exposure categories were associated with higher odds of CVV exposure (Table 2). This variable was collinear with age and although it remained significant when incorporated into multivariable models, the model that included age was retained instead of housing, based on a lower AIC value that indicated better performance.

The final mixed model identified three risk factors associated with higher odds of being exposed to CVV: sheep housing within 1 km of wetlands, a lake or pond; flock size; and age (Table 3). Ewes housed near wetlands, a lake or pond had 5.8-fold increased odds (95% CI: 1.4–23.8) of being exposed to CVV compared to ewes not housed near these landscape features. Smaller flocks had greater odds (OR: 7.3, 95% CI: 1.5–35.7) of being CVV seropositive in comparison to large flocks. With each additional year, the odds of CVV exposure at some point in the ewe’s lifetime increased 1.6-fold (95% CI: 1.3–2.0). The automated backward stepwise regression corroborated results from the manual process. The final model demonstrated that most of the variance was at the sheep level, although the farm effect was still important (farm variance = 27.5%).

**Table 3:** Risk factors associated with CVV seropositivity in sheep based on a final mixed logistic regression model with a random effect for farm of serological and questionnaire data obtained from sheep producers in Ontario, Canada (June–August 2021, n = 364).

Predictor variable		OR (95% CI)	P-value
**Age at sampling (years)**		1.63 (1.34–1.98)	<0.001
**Wetlands, lake, pond within 1 km**			
	No	Referent	–
	Yes	5.76 (1.40–23.77)	0.015
**Flock size (replacement ewes)**			
	Small: 20–140	7.26 (1.48–35.74)	0.015
	Medium: 141–399	4.75 (0.92–24.59)	0.063
	Large: ≥ 400	Referent	–
**Random intercept**		**Variance (95% CI)**	
**Farm**		27.5% (12.1%-51.1%)	

Evaluation of the BLUPs confirmed homoscedasticity and a relatively normal distribution. Upon examining the Pearson’s residuals, several moderate outliers were detected (from a single farm). However, when performing multivariable mixed regression without those outlying ewes, the results did not significantly differ and so the outliers remained in the dataset.

## Discussion

CVV is considered endemic to North America, yet little is known regarding exposure and disease in sheep. Widespread exposure had previously been illustrated in Yucatan, Mexico, the continental states of USA, and Saskatchewan, Canada [23, 33, 34]. This study illustrates a similar picture for Ontario, Canada, where one in three sheep at participating farms were positive for CVV exposure. Shepherds should therefore be cognizant of the risks posed when breeding ewes during mosquito season as exposure is highly likely.

Given that the annual number of market lambs in Ontario has been steeply increasing over the past decade [35], it is not surprising that CVV outbreaks in Ontario sheep flocks are being reported more regularly [20–22]. This study highlights three factors associated with greater risk of CVV exposure in ewes, which may be helpful when designing farm-level management factors to reduce risk of exposure and/or disease.

With each additional year, ewes are at 1.6 times greater risk of being exposed to CVV. Older ewes have had more opportunities to be bitten by a CVV-infected mosquito and thus exposed to the virus. Less than 10% of sampled ewes under two years of age had been exposed to CVV compared with 100% of ewes that were nine and older. For shepherds, it is likely difficult to utilize this finding to make management adjustments. Certainly, first breeding could be delayed until an ewe lamb has had at least one season of potential mosquito exposure and thus is more likely to have protective antibodies. However, exposure is not guaranteed and delaying breeding further would have economic consequences.

Ewes raised near wetlands, lakes, or ponds were found to be at greater risk of exposure than ewes not housed near these natural bodies of stagnant water. Stagnant water sources are prime breeding habitat for CVV vectors including *Anopheles punctipennis*, *Aedes vexans*, *Coquillettidia perturbans*, *Ochlerotatus (Oc*.*) canadensis* and *Oc*. *trivittatus* [12, 13]. These habitats likely contribute to higher abundance of mosquitoes near the farm and thus higher likelihood of CVV exposure. Removal of, or alterations to, these natural features is not ideal. Shepherds of flocks in these areas may need to consider methods of mosquito control, such as insecticide use to eliminate mosquito populations, tile drainage, brush maintenance, and elimination of outdoor water basins to deter future populations of mosquitoes [36]. However, this approach comes with a trade-off as ewe immunity would be reduced due to limited natural exposure to the virus and thus may inadvertently increase the risk of CVV disease.

Ewes in small flocks had higher odds of being seropositive for CVV compared to large flocks. It is unknown what mechanism drives this association. It is possible that flock size, either fully or in part, is acting as a proxy variable, potentially for other common exposures shared between the farms. Further research with a larger sample size would help clarify the relationship between flock size and CVV seropositivity. It is important to note that the two seronegative flocks in this study were among the largest flocks sampled, and they were also homogeneously young (≤ 2 years old) and located within the same provincial region.

While the study was not able to demonstrate any association between housing styles and CVV exposure risk, given that housing likely influences exposure to mosquitoes and it can be more easily manipulated than ecological components, this risk factor should continue to be explored in future research. A longitudinal cohort study of sheep naïve to CVV exposure and housed in one of three locales (i.e., closed barn, drylot, or pasture) that remained fixed over time would support a controlled setting for investigating associations between housing and CVV exposure.

There were several limitations in this study that are important to address. Farm participation was voluntary, possibly resulting in volunteer bias (a type of selection bias). Great effort was taken to advertise the study across multiple mediums. Still, farms that experienced CVV disease in their flocks may have shown greater interest in participating in the study, thereby resulting in an over-estimation of prevalence in Ontario. Misclassification bias may have also occurred, especially for the landscape and housing variables. All landscape variables were categorized by the shepherd and then verified by the researchers upon farm visit. However, flood plains exist alongside woodlots of varying sizes and nearby natural sources of water, both stagnant and flowing, and may vary by season and year. Future work would benefit by incorporating objective and reproducible landscape categories, such as a standardized vegetative index. Housing was highly variable and at times, inconsistent (as described above). Efforts were made to capture this variability through two different types of risk factors for housing, but it is possible neither variable sufficiently captured the housing effect. The sample size was also a key limitation. While the minimum target number of participating farms was achieved for the prevalence estimate, the number of ewes on different farms sampled was slightly below our target for risk factor analyses. Moreover, there were many variables included for exploration. A larger number of ewes sampled via additional farms would have increased the power to explore these associations, including interactions between variables.

## Conclusion

In conclusion, CVV exposure is common among sheep farms in southern Ontario. Ewes that are older, housed near natural stagnant water sources and part of smaller flocks are more likely to be seropositive. Since the risk of disease is associated with exposure of naïve sheep bred during mosquito season, especially in August and September when viral activity is the highest, Ontario sheep producers may benefit from breeding younger ewes outside of this higher risk period or modifying management practices during this period to limit outdoor exposure. This study can also be used as a guide for targeting mosquito surveillance and public health education for mitigating human exposure.

## Supporting information

S1 File Questionnaire administered to participating sheep producers. (PDF)

S2 File Description of serum testing protocol for CVV antibody detection. (PDF)

S3 File Causal diagram used to guide regression analysis. (PDF)
