# Supplementary material for: Cache a Killer: Cache Valley virus seropositivity and associated farm management risk factors in sheep in Ontario, Canada
Source: PLoS One. 2023 Aug 24;18(8):e0290443. doi: 10.1371/journal.pone.0290443 (PMC10449202; doi:10.1371/journal.pone.0290443)
Supplement: S1 File — (PDF) [file pone.0290443.s001.pdf]

Supplemental Document 1. Questionnaire administered to participating shepherds via telephone in advance of field sampling, summer 2021.

District #: [______] **Address: [____]**

# Street **Town** **Postal**

Note: Upon completion of all baseline surveys, each farm will be reassigned a new id through a random number generator to maintain anonymity during analysis and reporting of findings.

I. Sociodemographic

1. How many **years** have you farmed sheep? Check the best answer:

   < 2 [___] 2-5 [___] 6-10 [___] 11-20 [___] > 20 [___]

➢ **The remainder of this questionnaire refers specifically to <u>this</u> sheep farm enterprise.**

Sheep/Farm demographic data

II. Farm Site – this section is high level and focuses on your overall farm operation

1. To the best of your knowledge, how many **years** have sheep been continuously on this farm? Check the best answer:

   < 2 [___] 2-5 [___] 6-10 [___] 11-20 [___] > 20 [___]

2. What is the purpose for these sheep? Please check all that apply, and label "**1**" for primary purpose.

Meat	Milk/ Cheese	Wool	Breeding Sales	Show	Vegetation management	Research	Lifestyle	Other, describe:
								________

3. What breed(s) (purebred and crosses) of sheep do you currently have on the farm?

4. For each breed, how many sheep are in your **breeding** flock? Please distinguish between rams and ewes?

   i. Over the next 2 years, how do you anticipate the breeding flock will change in size?
      Circle the best answer choice:

**Decrease in size**	**Stable – no change**	**Increase in size**

5. Among your breeding ewes (all breeds), how many ewes are:

   <1 yo [____]   1-4 yo [____]   >4 yo [____]

6. Excluding sheep, identify other livestock raised on your farm. Mark "**X** " for all that apply.

Goats	
Beef cattle	
Dairy cattle	
Alpacas	
Llamas	
Horses	
Pigs	
Chickens	
Turkeys	
Other, please specify:	

7. Considering the last 2 years, which best describes your farm for each category below (A-D)?
   Please circle all that apply:

A.

**Livestock**	**Mixed-Crop/Livestock**

B.

**Certified organic**	**Uncertified organic**	**Conventional**

C.

**Commercial-family run**	**Commercial-industrial operation**	**Hobby/Lifestyle**

D.

**Farm adequately described above**	**Other, please describe your farm:** ______________________________

8. How many **acres** do you farm in total (including buildings, pasture, crops, wood lots and fallow land (i.e. land not in production))? ________acres *numeric input only*

   i. How many **acres** total do your sheep graze at any time during the year, including pastures and crops, either pre- or post-harvest? ________acres *numeric input only*

   ii. How many **acres** of woodlot do you have on your farm? _______acres (input 0 if you have none)

If value above >0, then proceed with questions below…

1. **Please identify location(s) on the MAP.**

2. Do your sheep have any access to these woodlot areas at any time during the year?

   Circle best answer:

Yes	No	Adjacent to woodlot/forest

III. Sheep Management – this section is about the management of your breeding flock (ewes only)

1. **Identify all locations on the MAP where sheep may reside throughout the year.**

2. Considering that different seasons require different management practices for your sheep, please designate which season you consider each month when answering the questions in this section, whereby:

Winter = **W** Spring = **G** Summer = **S** Fall = **F**

Jan	Feb	Mar	Apr	May	Jun	Jul	Aug	Sep	Oct	Nov	Dec

3. For the last 2 years, please describe how your sheep are managed in each season.

In section **A** below, for each season (W, G, S, F), mark with an "**X**" the management styles used. If multiple categories apply within a season, then allocate the appropriate percentage to each category such that they total to 100% for the season.

A.

**Management style:**	**W**	**G**	**S**	**F**
Pasture / Grazing				
Dry lot – **no** shelter **nor** grazing access				
Barn with dry lot access – **no** grazing				
Total barn confinement – **no** grazing, **no** dry lot access				
Other, please specify:				

In section **B** below, for each season (W, G, S, F), mark with an "**X**" all available water sources, regardless if sheep actually use the water source(s).

B.

**Available water sources:**	**W**	**G**	**S**	**F**
Pond				
Natural stream/river				
Water tank/trough				
Automatic water system, e.g. water bowls				
Other, please specify:				

i. If a water tank/trough is used in any season, please describe (1) the cleaning process and (2) the frequency of that cleaning?

1. Cleaning process:
2. Cleaning frequency: *input numeric value only*

4. For <u>each</u> season, where are your sheep located during the following periods?
   Please mark each location that applies with an "**X**".

**WINTER**	Dawn	Day	Dusk	Night
Pasture / Grazing				
Dry lot – **no** shelter **nor** grazing access				
Barn with dry lot access – **no** grazing				
Total barn confinement – **no** grazing **nor** dry lot access				
Other, please specify:				

**SPRING**	Dawn	Day	Dusk	Night
Pasture / Grazing				
Dry lot – **no** shelter **nor** grazing access				
Barn with dry lot access – **no** grazing				
Total barn confinement – **no** grazing **nor** dry lot access				
Other, please specify:				

**SUMMER**	Dawn	Day	Dusk	Night
Pasture / Grazing				
Dry lot – **no** shelter **nor** grazing access				
Barn with dry lot access – **no** grazing				
Total barn confinement – **no** grazing **nor** dry lot access				
Other, please specify:				

**FALL**	Dawn	Day	Dusk	Night
Pasture / Grazing				
Dry lot – **no** shelter **nor** grazing access				
Barn with dry lot access – **no** grazing				
Total barn confinement – **no** grazing **nor** dry lot access				
Other, please specify:				

For the following two questions, please identify locations on the MAP as well.

5. On and surrounding your farm (within ~1 km), please check all applicable scenarios for unintended water holding locations, and **identify on the map**:

**Stagnant water sources:**	Mark **X** if applicable:
Tree holes	
Discarded tires	
Retention basins	
Drainage ditches	
Wetlands	
Ponds	
Pools [seasonally unused]	
Fountains with stagnant water	
Outside containers (flowerpot, buckets, etc.)	
Other, please specify:	

6. In the last 2 years, where on the farm did you have any issues with mosquitoes in any capacity (e.g., hovering around sheep flocks, concentrated near drains or gutters, difficulty working outdoors, etc.)?
If not applicable, write " NA ". Otherwise, please describe and **Identify on the MAP.**

7. Which month(s) do you normally shear your sheep? Circle all that apply.

Jan	Feb	Mar	Apr	May	Jun	Jul	Aug	Sep	Oct	Nov	Dec

IV. Sheep Movement

1. Were any replacement ewes born offsite? Circle the best answer choice.

Yes	No

   a. If yes, please also provide additional details below, including location of birth and arrival date on this farm.

2. In the last 2 years, have any of your breeding flock spent time temporarily off your farm or a property managed by you? Circle the best answer choice.

Yes	No

   a. If yes, please also provide additional details below.

**Offsite location:**	**W**	**G**	**S**	**F**
Loaning of a ram(s) to another farm				
Attend a fair				
Shared pasture with another flock				
Other, please specify:				

V. Breeding / Lambing Practices

1. During the breeding and lambing periods, where are the breeding ewes managed?

   Please circle **P** for ONLY RELEVANT MONTHS for 1st time ewe lambers ("primiparous");

   Please circle **M** for ONLY RELELVANT MONTHS for ewes that have lambed at least once prior ("multiparous").

**Breeding period:**	**J**	**F**	**M**	**A**	**M**	**Jn**	**Ju**	**A**	**S**	**O**	**N**	**D**
Pasture / Grazing	P M	P M	P M	P M	P M	P M	P M	P M	P M	P M	P M	P M
Dry lot – **no** shelter **nor** grazing access	P M	P M	P M	P M	P M	P M	P M	P M	P M	P M	P M	P M
Barn with dry lot access – **no** grazing	P M	P M	P M	P M	P M	P M	P M	P M	P M	P M	P M	P M
Total barn confinement – **no** grazing, **no** dry lot access	P M	P M	P M	P M	P M	P M	P M	P M	P M	P M	P M	P M
Other, please specify:	P M	P M	P M	P M	P M	P M	P M	P M	P M	P M	P M	P M
**Lambing period:**	**J**	**F**	**M**	**A**	**M**	**Jn**	**Ju**	**A**	**S**	**O**	**N**	**D**
Pasture / Grazing	P M	P M	P M	P M	P M	P M	P M	P M	P M	P M	P M	P M
Dry lot – **no** shelter **nor** grazing access	P M	P M	P M	P M	P M	P M	P M	P M	P M	P M	P M	P M
Barn with dry lot access – **no** grazing	P M	P M	P M	P M	P M	P M	P M	P M	P M	P M	P M	P M
Total barn confinement – **no** grazing, **no** dry lot access	P M	P M	P M	P M	P M	P M	P M	P M	P M	P M	P M	P M
Other, please specify:	P M	P M	P M	P M	P M	P M	P M	P M	P M	P M	P M	P M

2. How many **days** are the rams kept with the ewes during the breeding season? __________days
   numeric input only

3. Estimating from the previous breeding season, please answer the questions within the table (A-C).

Note "primiparous" refers to ewe lambs, and "multiparous" refers to ewes that have given birth at least once prior.

		**Primiparous**			**Multiparous**		
**A.**	How many ewes were exposed to rams?						
**B.**	Of these ewes, how many became pregnant and lambed?						
**C.**	Which category describes your satisfaction with this fertility rate? Circle the best choice among the 3 options.	Better than expected	As expected	Worse than expected	Better than expected	As expected	Worse than expected

4. Did the ewes start to lamb when expected (i.e., approximately 142-148 days after ram introduction)? Circle the best answer choice.

**Yes**	**No – earlier than expected**	**No – later than expected**

i. If No, please comment on why you believe such may have occurred?

5. As of today, in your current breeding flock…
How many ewes have not yet lambed for the first time? ___________ *numeric input only*

i. Of these ewe lambs… *numeric input only*

1. How many are currently pregnant?	
2. How many were exposed to the ram but have not been pregnancy-checked?	
3. How many were not yet exposed to the ram but intend to be exposed this season?	

6. What is the average age (in **months**) you normally first expose ewe lambs to rams for breeding?

__________months old *numeric input only*

7. Please identify all the times you routinely body condition score (BCS) your breeding ewes.

Do NOT include events when you suspected an issue with an individual or group of ewes.

Circle all categories that apply.

P stands for ewe lambs, and **M** stands for mature lambs.

Prebreeding	P	M
Mid gestation	P	M
Lambing	P	M
Weaning	P	M
Other, please specify:	P	M

VI. Disease

1. In the last 2 years, how many of the following scenarios occurred in your breeding flock? Please answer both for 1st time ewe lambers ("**P**") and for ewes that gave birth at least once prior ("**M**")?

**Birth abnormalities**	**P**	**M**	**Total Breeding Ewes at the Time:**
**a.** How many ewes suffered dystocias?			
**b.** How many ewes aborted?			
**b.i.** If a diagnosis was made, can you please provide the diagnosis and how it was determined:			
	**P**	**M**	**Out of How Many Total Lambs Born:**
**c.** How many lambs were born with any kind of deformity?			
**d.** How many lambs were delivered dead (i.e. stillborn)?			
**e.** How many lambs were delivered with twisted/fixed joints, twisted spine, and/or humped?			
**f.** How many lambs died within 1st week of life?			

****

WARNING – please note that a picture appears on the next page of a lamb diagnosed with Cache Valley Virus, which may be disturbing to look at. We apologize for any discomfort caused, but we wanted to provide a reliable image to assist you in answering the questions to come.

Cache Valley virus is a mosquito-borne virus that causes severe congenital deformities in newborn lambs.

Have you ever had a diagnosis of Cache Valley Virus (CVV) affected lambs in your flock?

If yes, please continue with the subset of questions for # 2.

Here is a picture of a classic example:

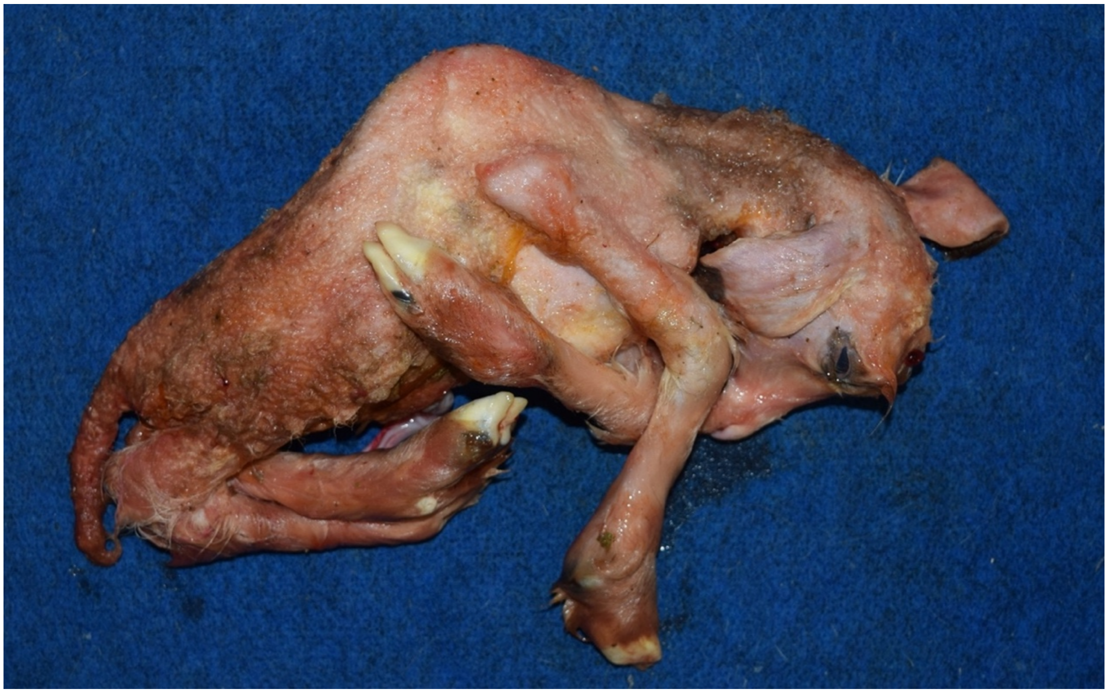

Figure 1. This lamb shows the classic deformities associated with Cache Valley virus infection in early pregnancy. Note the limbs are twisted and the joints fixed (**arthrogryposis**); the spine is twisted (**kyphosis**) and humped (**lordosis**). Additionally, the brain of the lamb may be underdeveloped. Affected lambs are usually born at term but are either stillborn or die soon after birth. Lambs infected with the virus in very early pregnancy may die and be resorbed; lambs infected in later pregnancy are born normal. *Photo courtesy M. Spinato AHL, University of Guelph*

2. How were the cases diagnosed? Mark each applicable category with an "**X**".

Veterinarian confirmed that deformities were consistent with CVV infection.	
Diagnostic lab confirmed CVV infection on a lamb submitted for testing.	
Deformities were consistent with what I have read, seen or heard about CVV.	
Other, please describe:	

3. For <u>each</u> lambing period that CVV infected the breeding flock, please complete the table below. For the outbreak date, fill in the year and month the outbreak started using the format YYYY-MM. Note that "**P**" stands for 1st time ewe lambers and "**M**" stands for ewes that gave birth at least once prior.

For example, if in January 2016, 50 ewes (of which 20 were 1st time ewe lambers) delivered 80 lambs (of which 36 were delivered by 1st time ewe lambers), and 3 exhibited deformities consistent with CVV (of which 2 were delivered by 1st time ewe lambers), then we would complete the table as such:

Example:

**Date of outbreak:** 2 0 1 6 - 0 1	**P**	**M**
a. How many ewes total delivered lambs?	20	30 (= 50 - 20)
ii. How many lambs total were born to those ewes?	36	44 (= 80 – 36)
b.i. How many of those lambs showed deformities consistent with CVV?	2	1 (= 3 – 2)

Now for you to complete:

**Date of outbreak:** Y Y Y Y - M M	**P**	**M**
a. How many ewes total delivered lambs?		
b. How many lambs total were born to those ewes?		
b.i. How many of those lambs showed deformities consistent with CVV?		

i. Were any of the affected CVV lambs born with affected littermates, yes/no? ___________

ii. Were any of the affected CVV lambs born with normal appearing littermates, yes/no? ____

4. Are you aware of a specific ewe giving birth to CVV infected lambs at more than one lambing?

i. Is there anything else noteworthy about this/these ewes? ___________________________

VII. Climate – this next category of questions aims at capturing climate patterns on your farm

1. How would you categorize each of the following scenarios on your farm over the last 5 years? Please circle the appropriate symbol (e.g. increase, decrease, or no change) for questions A-E for each of the four seasons.

	**Scenario:**	**Winter**	**Spring**	**Summer**	**Fall**
**A.**	**Precipitation**	↑ ↓ —	↑ ↓ —	↑ ↓ —	↑ ↓ —
**B.**	**Extreme/unusual temperature fluctuations**	↑ ↓ —	↑ ↓ —	↑ ↓ —	↑ ↓ —
**C.**	**Frequency of unusual/intense storms**	↑ ↓ —	↑ ↓ —	↑ ↓ —	↑ ↓ —
**D.**	**Localized seasonal flooding with standing water**	↑ ↓ —	↑ ↓ —	↑ ↓ —	↑ ↓ —
**E.**	**Mosquito populations**	↑ ↓ —	↑ ↓ —	↑ ↓ —	↑ ↓ —

2. In regard to the growing season over the last 5 years, please identify any changes:

	**Length**	**Start date**	**End date**	**Overall variability**
**Growing season**	↑ ↓ —	↑ ↓ —	↑ ↓ —	↑ ↓ —

i. If there is anything else you would like to comment on about your growing season, please share.

3. Do you use any strategies to hasten drainage of standing water that may be caused by seasonal flooding? Circle best answer:

Yes	No

i. If yes, please describe (e.g. tile draining land, pumping, ditches deepened to facilitate flow):

VIII. Insecticides / Repellents

1. What strategies have you used over the last 2 years to control mosquitoes and flies?

	Animals	Structures/Areas	People
**Product name**			
**Formulation**			
**Application method**			
**Total dispensed amount per treatment**			
**Application frequency** (e.g. multiple times/day, daily, weekly, etc.)			
**Location(s)**			
**Which sheep if animal based** (e.g. lambs, breeding ewes, rams, etc.)		**N/A**	**N/A**

May I take a picture of each of the products?

2. Anything else that you would like to tell us about the mosquitoes on your farms?

IX. Wildlife Sightings

1. Over the last 2 years, have you observed deer or evidence of deer on your farm?
   To complete the table, for each season (1-4), circle 1 of the 5 frequency categories for each of the 3 times of day (a-c).
   Note that **Rare =** ≤ 3x per month; **Occasional** = ~1x per week; **Frequent** = >1x per week.

#	Season	Time of Day	Frequency of Deer Sightings				
1.	Winter	a. Dawn	Never	Rare	Occasional	Frequent	Do not know
		b. Day	Never	Rare	Occasional	Frequent	Do not know
		c. Dusk / Night	Never	Rare	Occasional	Frequent	Do not know
2.	Spring	a. Dawn	Never	Rare	Occasional	Frequent	Do not know
		b. Day	Never	Rare	Occasional	Frequent	Do not know
		c. Dusk / Night	Never	Rare	Occasional	Frequent	Do not know
3.	Summer	a. Dawn	Never	Rare	Occasional	Frequent	Do not know
		b. Day	Never	Rare	Occasional	Frequent	Do not know
		c. Dusk / Night	Never	Rare	Occasional	Frequent	Do not know
4.	Fall	a. Dawn	Never	Rare	Occasional	Frequent	Do not know
		b. Day	Never	Rare	Occasional	Frequent	Do not know
		c. Dusk / Night	Never	Rare	Occasional	Frequent	Do not know

A sincere thank you for your time and input. We will be back in touch with our findings. If at any time you want to follow up with additional comments or questions, please contact Michele Bergevin by email at mberg@uoguelph.ca

[1] Mosquito flight range estimated from the following resource:
Verdonschot, P., and Besse-Lototskaya, A. (2014). Flight distance of mosquitoes (Cuclicidae): A metadata analysis to support the management of barrier zones around rewetted and newly constructed wetlands. *Limnologica,* 45: 69-79.
