# Supplementary material for: Cache a Killer: Cache Valley virus seropositivity and associated farm management risk factors in sheep in Ontario, Canada
Source: PLoS One. 2023 Aug 24;18(8):e0290443. doi: 10.1371/journal.pone.0290443 (PMC10449202; doi:10.1371/journal.pone.0290443)
Supplement: S3 File — (PDF) [file pone.0290443.s003.pdf]

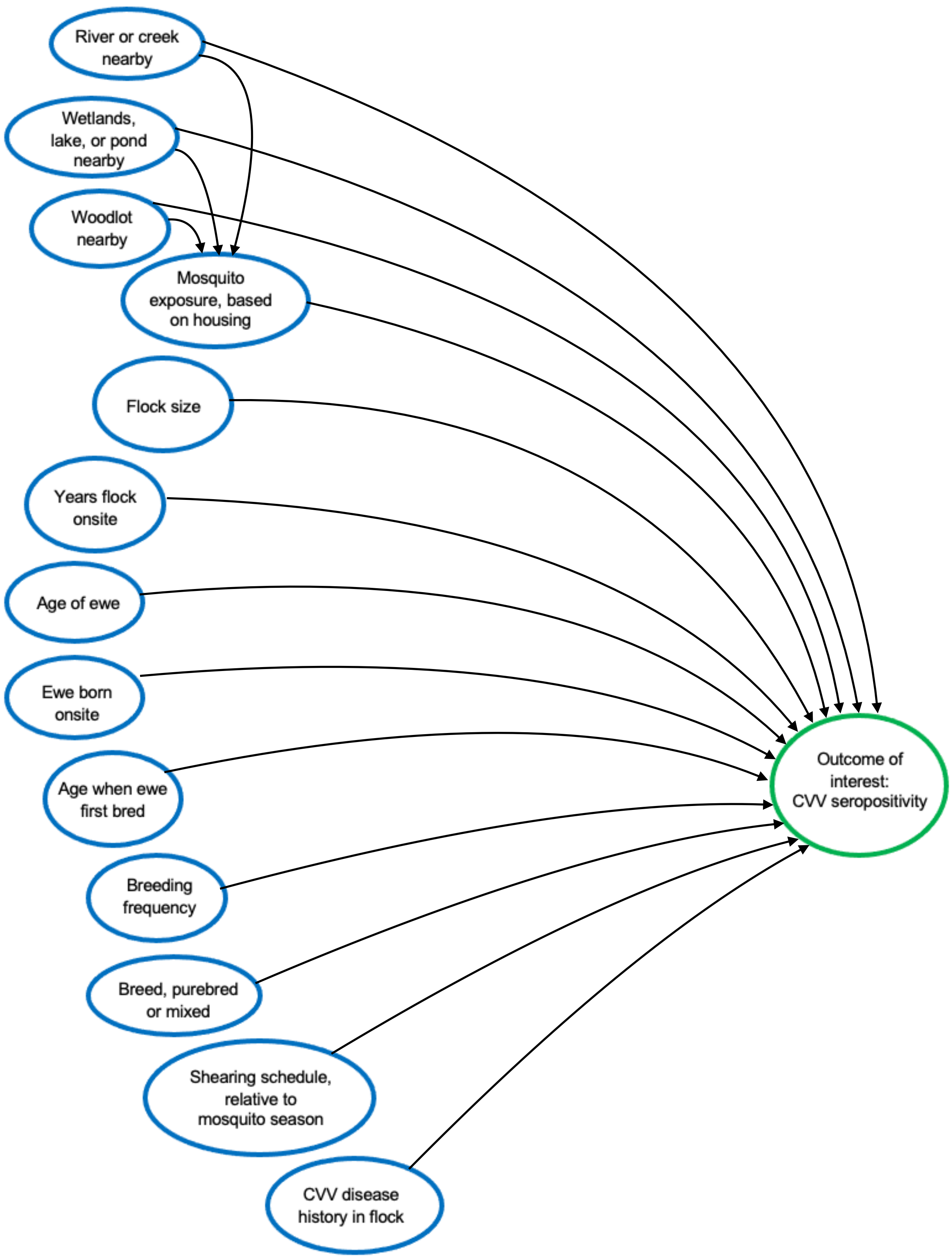

Supplemental document 3. Causal diagram depicting the associations between farm management, ewe characteristics, and surrounding farm environment (risk factors, blue circles) and ewe CVV seroprevalence (outcome of interest, green circle).
